# The variability of nuclear DNA content of different *Pelargonium* species estimated by flow cytometry

**DOI:** 10.1371/journal.pone.0267496

**Published:** 2022-04-28

**Authors:** Sylvia Plaschil, Simone Abel, Evelyn Klocke

**Affiliations:** Julius Kühn Institute (JKI)—Federal Research Centre of Cultivated Plants, Institute for Breeding Research on Horticultural Crops, Quedlinburg, Germany; Leibniz-Institute of Plant Genetics and Crop Plant Research (IPK), GERMANY

## Abstract

*Pelargonium* is a versatile genus mainly from the Cape Region, South Africa. The genus is divided into four subgenera and 16 sections characterized by several groups of chromosomes sizes and numbers. The DNA content of species from all subgenera and sections of *Pelargonium*, except for the sections *Subsucculentia* and *Campylia* was estimated using flow cytometry. Nuclei of *Pelargonium* samples (leaf or petal tissue) and an internal plant standard (leaf tissue) were isolated together and stained with propidium iodide. The DNA content was estimated providing that the 2C peaks of sample and standard be in linearity in the flow cytometer histograms. In total, 96 *Pelargonium* accessions of 60 species (22 *Pelargonium* species for the first time) were analyzed. The 2C DNA content ranged from 0.84 pg (*P*. *longifolium*, section *Hoarea*) to 6.69 pg (*P*. *schizopetalum*, section *Magnistipulacea*) and the corresponding 1C*x* DNA content from 0.42 pg (*P*. *longifolium*) to 1.72 pg (*P*. *transvaalense*. This demonstrates the high plasticity within the genus *Pelargonium*. Some species, such as *P*. *peltatum* accessions revealed a pronounced endopolyploidization in leaves but not in petals underlining the importance to choose the right tissue as sample for the flow cytometry analysis. The reported genome sizes are a step forward towards the characterization of the *Pelargonium* collection within the German Gene Bank for Ornamental Plants and a valuable base for future sequencing programs of the *Pelargonium* genomes.

## Introduction

Pelargoniums are famous bedding and balcony plants around the world. *Pelargonium* and *Geranium* were a common genus until the 18^th^ century. The remarkable seed shape remembering a cranesbill (crane in Greek: geranos) was the defining feature equal for both. However, the flower architecture is very different: Species of genus *Geranium* show actinomorphic flowers whereas *Pelargonium* plants have zygomorphic ones. Thus, *Geranium* and *Pelargonium* have been divided into two genera of the family Geraniaceae [1]. Perhaps the long uninterrupted popularity of *Pelargonium* plants is the reason why the term geranium persists not only in English-speaking countries even in scientific publications. For the sake of correctness, we only use the term *Pelargonium*.

The genus *Pelargonium* comprised about 280 taxa [2]. Pelargoniums are mainly distributed in the Cape Region of South Africa [3]. This region is distinguished as a hotspot for its plant diversity and endemism due to very different climate and geographical conditions in a relatively small area. The exceptional climatic stability during the Pleistocene is accepted as a further major factor promoting the abundance of plant species in this region [4]. Species of the genus *Pelargonium* colonize very different habitats and differ greatly in morphology, anatomy, and cytology. The high number of habits found in *Pelargonium* probably resulted from the nested radiation in Winter-rainfall region occurred in response to aridification in the mid-Miocene and to the ensuing fragmentation of niches [5–7].

Considering the high diversity, the genus is arranged in sixteen infrageneric sections [2, 8] of four subgenera [2]. New *Pelargonium* species are continuously being described [e.g. 9–11]. In addition to diverse morphological features, chromosomes of different sizes were found in the respective sections and species [12–15]. Extensive phylogenetic analyses were performed using various molecular methods [8, 16–18]. The remarkable high levels of organelle genomic rearrangements were investigated and phylogenetic analyses on this base confirmed the subgeneric structure of two main clades (small and large chromosome clade) and five subclades [7, 19]. Although monophyly has already been demonstrated for some sections as *Ligularia* and *Hoarea*, more molecular investigations are necessary to prove this for the other sections as well [7]. In addition, the crown node age for the *Pelargonium* was dated to 9.7 Mya (Late Miocene) [7]. The accelerated mitochondrial substitution rates and the exceptional variability in the plastome are further outstanding traits of the genus *Pelargonium* and are the subject to intense research [20–29].

First pelargoniums came to Europe as early as 1600. *Pelargonium* species have long been collected in botanical gardens. Nowadays, breeders keep *Pelargonium* collections as a resource for further crossbreeding to gain a greater genetic variability of the cultivars. Despite a long breeding history, the genetics of commercial cultivars is limited. In Germany, a “subnetwork *Pelargonium*” was established in the very last years. It belongs to the German Gene Bank for Ornamental plants (DGZ) [30]. The foundation of the DGZ aims to preserve the diversity of ornamental plant genetic resources including *Pelargonium* and allows the long-term use of these resources. The *Pelargonium* collection at Julius Kühn Institute (JKI) is a part of it. The collection consists of *Pelargonium* species and accessions that have been kindly made available by German breeders’ houses over the last twenty years. We started to characterize the *Pelargonium* JKI collection more in detail. Several questions arose regarding the correct botanical classification. To clarify it and to provide more information about the comprehensive *Pelargonium* JKI collection, we determined the DNA content of the species / accessions using the flow cytometry (FCM).

First, Greilhuber [31] determined a 1C DNA content of 8.1 pg for *P*. *radula* by Feulgen cytophotometry. Since the 1980s with the improved equipment of the laboratories, FCM with plant cells developed into a widely used method for determining the plant DNA content [32]. However, DNA amounts in pelargoniums have rarely been determined so far. The values published by different authors differ considerably. For *P*. x *hortorum* L.H. Bailey, which traces back to *P*. *zonale* and is the most important commercial *Pelargonium* cultivar group, Cassells *et al*. [33] stated a 2C DNA content of 3.16 pg while Weng *et al*. [22] announced a content of 1.79 pg for *P*. *zonale*. Nieuwenhuis [34] registered also large differences between his and Weng *et al*.’s [22] 2C DNA contents for the same *Pelargonium* species.

In the present paper, we used FCM for the determination of the 2C DNA content in a collection of 60 *Pelargonium* species and overall 96 accessions. For 22 *Pelargonium* species, the DNA content was determined for the first time. The investigations should provide information about intraspecific and intrasectional variability of genome size in *Pelargonium*, help to detect polyploid accessions and support further breeding efforts. With known ploidy the 1C*x* content, a valuable feature of the genome, was estimated to prove the hypothesis of genome up- or downsizing in the genus *Pelargonium* [34, 35]. Moreover, experimental challenges of FCM and reasons for very different published DNA contents of *Pelargonium* species are discussed.

## Materials and methods

### Plant material

Samples for plant DNA flow cytometry were taken from greenhouse plants. The *Pelargonium* JKI collection consists of 60 *Pelargonium* species from fourteen sections. More than one accession / subspecies was tested out of 23 species. To maintain healthy young plants, the stock plants are regularly propagated using cuttings. Each accession is represented by at least three plants (Table 1), [S1 Table]. As internal standards a *Raphanus sativus* L. accession, provided by the Leibniz Institute of Plant Genetics and Crop Plant Research Gatersleben, Germany (Ra) (2C = 1.11 pg) [36] and *Solanum lycopersicon* L. ‘Stupické’ (To) (2C = 1.96 pg) [36] were used. *Brassica oleracea* var. *botrytis* L. (cauliflower) ‘Korso’ (Ca) (2C = 1.31 pg) [37] also served as a standard for *P*. *grandiflorum*. The standards are kept as *in vitro* plants on solid medium MS [38] supplemented with 0.2 mg L^-1^ 1-naphthalene acetic acid, 3% sucrose in a climatic chamber (25°C, 16 hours light / 8 hours dark).

**Table 1:** *Pelargonium* species / accessions with JKI collection number for which the 2C DNA content was determined in this study. The assignment of sections is based on Röschenbleck *et al*. [2]. Chromosome numbers, ploidy levels and chromosome lengths were obtained from the literature. 1C*x* DNA contents in brackets were calculated with presumable ploidy level (bold). SD: standard deviation, Ra: *Raphanus*, To: tomato, Ca: cauliflower.

Subgenus	Species	№ JKI Accession	Chromosome size (μm)	№ of chromo-somes (n)	Ploidy level (*x*)	2C DNA content (pg)	SD	1Cx DNA content (pg)	Internal standard	№ of samples
*Magnipetala*	Section ***Chorisma***									
	*P*. *mollicomum*	131	> 1.5	11	2	2.53	0.10	1.26	Ra	11
	*P*. *tetragonum*	30	1.8–2.5	11	2	2.91	0.05	1.46	Ra	4
	*P*. *worcesterae*	72	> 1.5	11	2	2.63	0.14	1.32	Ra	6
	*Section* ***Jenkinsonia***									
	*P*. *mutans*	133	> 1.5	9	2	2.98	0.01	1.49	Ra	3
	*P*. *mutans*	316	> 1.5	9	2	2.74	0.00	1.37	To	3
	*P*. *trifidum*	147	> 1.5	9	2	2.37	0.06	1.18	Ra	4
	Section ***Myrrhidium***									
	*P*. *myrrhifolium* var. *myrrhifolium*	20	> 1.5	11	2	2.16	0.10	1.08	Ra	11
	*P*. *myrrhifolium* var. *coriandrifolium*	22	> 1.5	11	2	1.55	0.05	0.77	Ra	10
	*P*. *myrrhifolium* var. *synnotii*	21	> 1.5	11	2	1.53	0.07	0.76	Ra	6
*Parvulipetala*	Section ***Isopetalum***									
	*P*. *cotyledonis*	74	< 1.5	8	2	0.94	0.02	0.47	Ra / To	5
	*P*. *cotyledonis*	116	< 1.5	8	2	0.92	0.01	0.46	To	3
	Section ***Peristera***									
	*P*. *australe*	109	< 1.5	9	2	1.12	0.01	0.56	To	3
	*P*. *grossularioides*	13	< 1.5	8, 19	2, **4**	2.57	0.14	(0.64)	Ra / To	9
	*P*. *rodneyanum*	65	< 1.5			1.31	0.03	n.d.	Ra / To	5
	Section ***Reniformia***									
	*P*. *abrotanifolium*	101	< 1.5	8	2, **4**	3.35	0.13	(0.84)	Ra	5
	*P*. *ionidiflorum*	73	< 1.5	8	2	1.60	0.05	0.80	Ra	3
	*P*. *odoratissimum*	52	< 1.5	8	2	1.74	0.05	0.87	Ra	9
	*P*. *odoratissimum*	432	< 1.5	8	2	1.66	0.07	0.83	Ra / To	10
	*P*. *reniforme* subsp. *reniforme*	28	< 1.5	8	**2**, 4	1.69	0.06	(0.85)	Ra	9
	*P*. *sidoides*	142	< 1.5	8	2, **4**	3.58	0.05	(0.90)	Ra	4
	*P*. *sidoides*	321	< 1.5	8	2, 4	6.39	0.13	(0.80 ≙ **8*x***)	To	7
*Paucisignata*	Section ***Ciconium***									
	*P*. *acetosum*	1	1.5–2.7	9	2	2.34	0.06	1.17	Ra	9
	*P*. *acetosum*	1/7	1.5–2.7	9	2	2.44	0.05	1.22	Ra	10
	*P*. *acetosum*	102	1.5–2.7	9	2	2.45	0.06	1.22	Ra / To	8
	*P*. *acraeum*	103	1.6–2.6	9	2	2.47	0.02	1.24	To	3
	*P*. *alchemilloides*	2	> 1.5	8, 9, 17	2, **4**	4.23	0.14	(1.06)	Ra / To	11
	*P*. *alchemilloides*	104	> 1.5	8, 9, 17	2, **4**	4.24	0.15	(1.06)	Ra / To	9
	*P*. *aridum*	69	> 1.5	9	2	2.38	0.06	1.19	Ra	9
	*P*. *aridum*	106	> 1.5	9	2	2.34	0.09	1.17	Ra	4
	*P*. *frutetorum*	122	1.7–2.9	9	2	2.32	0.00	1.16	To	3
	*P*. *frutetorum*	46	1.7–2.9	9	2	2.40	0.02	1.20	To	3
	*P*. *inquinans*	15	1.7–2.6	9	2	2.44	0.04	1.22	To	4
	*P*. *inquinans*	128	1.7–2.6	9	2	2.37	0.03	1.19	To	3
	*P*. *multibracteatum*	18	> 1.5	9	4	3.59	0.15	0.90	Ra	5
	*P*. *multibracteatum*	132	> 1.5	9	4	3.57	0.09	0.89	Ra	8
	*P*. *peltatum*	26	1.6–2.6	9	**2**, 4	2.24	0.05	(1.12)	Ra	16
	*P*. *peltatum*	44	1.6–2.6	9	**2**, 4	2.22	0.06	(1.11)	Ra	17
	*P*. *peltatum*	135	1.6–2.6	9	**2**, 4	2.19	0.05	(1.10)	Ra	22
	*P*. *peltatum*	506	1.6–2.6	9	**2**, 4	2.19	0.05	(1.10)	Ra	18
	*P*. *quinquelobatum*	138	> 1.5	9	2	4.54	0.13	(1.14 ≙ **4*x***)	Ra	3
	*P*. *tongaense*	505	1.6–3.0	9	2	2.77	0.06	1.38	Ra	4
	*P*. *zonale*	33	1.7–3.2	9	2	2.30	0.05	1.15	Ra	4
	*P*. *zonale*	43	1.7–3.2	9	2	2.34	0.07	1.17	Ra	3
	*P*. *zonale*	149	1.7–3.2	9	2	2.40	0.05	1.20	Ra	4
	*P*. *zonale*	504	1.7–3.2	9	2	2.39	0.02	1.20	Ra	3
	*P*. *zonale*	508	1.7–3.2	9	2	2.39	0.01	1.20	Ra	3
	*P*. *zonale*	509	1.7–3.2	9	4	4.55	0.20	1.14	Ra / To	6
	**unassigned species**									
	*P*. *caylae*	47	1.6–3.0	9	4	4.85	0.07	1.21	To	4
	*P*. *caylae*	112	1.6–3.0	9	4	4.90	0.12	1.22	To	7
	*P*. *caylae*	318	1.6–3.0	9	4	5.00	0.06	1.25	To	5
	*P*. *transvaalense*	146	> 1.5	9	2	3.45	0.08	1.72	To	4
*Pelargonium*	Section ***Cortusina***									
	*P*. *cortusifolium*	115	< 1.5	11	2	1.15	0.04	0.58	To	5
	*P*. *echinatum*	10	< 1.5	11	2	1.09	0.03	0.54	To	4
	*P*. *echinatum*	119	< 1.5	11	2	1.06	0.02	0.53	To	4
	*P*. *magenteum*	130	< 1.5	11	2	1.13	0.03	0.57	To	5
	*P*. *magenteum*	433	< 1.5	11	2	1.30	0.00	0.63	To	6
	Section ***Hoarea***									
	*P*. *longifolium*	17	< 1.5	10–11	2	0.84	0.02	0.42	To	3
	*P*. *oblongatum*	23	< 1.5	11	2	1.77	0.06	(0.44 ≙ **4*x***)	Ra / To	8
	Section ***Ligularia***									
	*P*. *fulgidum*	11	< 1.5	11	2	1.54	0.06	0.77	Ra	8
	*P*. *fulgidum*	48	< 1.5	11	2	1.55	0.05	0.78	Ra	11
	*P*. *fulgidum*	123	< 1.5	11	2	1.50	0.05	0.75	Ra	8
	*P*. *hirtum*	14	< 1.5	11	**2**, 4	1.40	0.02	(0.70)	To	3
	*Section* ***Magnistipulacea***									
	*P*. *bowkeri*	425	< 1.5	11	4	2.89	0.06	0.72	Ra / To	3
	*P*. *schizopetalum*	141	< 1.5			6.69	0.16	n.d.	To	6
	Section ***Otidia***									
	*P*. *carnosum*	5	< 1.5	11	2	1.30	0.06	0.65	To	6
	*P*. *crithmifolium*	8	< 1.5	11	4	1.37	0.01	(0.69 ≙ **2x**)	To	5
	*P*. *klinghardtense*	16	< 1.5	11	2	1.38	0.02	0.69	To	4
	*P*. *laxum*	129	< 1.5	11	2	1.49	0.06	0.75	Ra / To	8
	Section ***Pelargonium***									
	*P*. *betulinum*	501	< 1.5	11	2	1.07	0.04	0.53	To	3
	*P*. *betulinum*	502	< 1.5	11	2	1.05	0.03	0.52	To	3
	*P*. *capitatum*	40	< 1.5	11	6	3.47	0.03	0.58	To	3
	*P*. *cordifolium*	6	< 1.5	11	2	1.06	0.02	0.53	To	3
	*P*. *cordifolium*	662	< 1.5	11	2	1.05	0.02	0.52	To	5
	*P*. *crispum*	657	< 1.5	11	2	1.10	0.02	0.55	To	3
	*P*. *cucullatum* subsp.?	9	< 1.5	11	2	1.15	0.01	0.58	To	5
	*P*. *cucullatum* subsp?	41	< 1.5	11	2	1.14	0.00	0.57	To	3
	*P*. *cucullatum* subsp?	118	< 1.5	11	2	1.10	0.03	0.55	To	6
	*P*. *fruticosum*	507	< 1.5	11	2	1.04	0.06	0.52	To	5
	*P*. *glutinosum*	124	< 1.5	11	4	2.32	0.07	0.58	Ra	5
	*P*. *grandiflorum*	12	< 1.5	11	2	0.99	0.02	0.49	Ra / Ca	3
	*P*. *grandiflorum*	125	< 1.5	11	2	0.95	0.01	0.48	Ca	3
	*P*. *graveolens*	126	< 1.5	10, 11	8	4.09	0.06	0.51	To	4
	*P*. *graveolens*	609	< 1.5	10, 11	8	4.04	0.04	0.50	To	5
	*P*. *graveolens*	666	< 1.5	10, 11	8	3.78	0.05	0.47	Ra / To	3
	*P*. *panduriforme*	134	< 1.5	11	4	2.13	0.01	0.53	Ra	3
	*P*. *papilionaceum*	25	< 1.5	11	4	2.26	0.08	0.56	Ra	6
	*P*. *quercifolium*	137	< 1.5	11	4	2.31	0.02	0.58	Ra	3
	*P*. *scabrum*	140	< 1.5	11	2	1.08	0.01	0.54	To	4
	*P*. *tabulare*	29	< 1.5	11	2	1.31	0.01	0.66	To	3
	*P*. *tomentosum*	144	< 1.5	11	4	2.26	0.02	0.56	Ra	3
	*P*. *vitifolium*	32	< 1.5	11	8	4.49	0.17	0.56	Ra / To	6
	*P*. *vitifolium*	39	< 1.5	11	8	4.24	0.10	0.54	Ra / To	7
	*P*. *vitifolium*	51	< 1.5	11	8	4.22	0.03	0.53	To	3
	Section ***Polyactium***									
	*P*. *pulverulentum*	136	< 1.5	11	**2**, 4, 6	0.89	0.02	(0.45)	To	3
	*P*. *radulifolium*	139	< 1.5	10, 11	6	4.29	0.18	0.72	Ra / To	12
	*P*. *triste*	421	< 1.5	10, 11	4, **6**	4.12	0.07	(0.69)	To	5

### Flow cytometry

Sample plant material was stored in a wet petri dish on ice until preparation according to a modified JKI protocol. Using nuclei extraction and staining buffer of the CyStain^®^ PI Absolute P Kit (Sysmex, Germany), propidium iodide (PI) (1 mg/1 mL, Sigma Aldrich), and ribonuclease A (1 mg/1 mL, Serva) samples were prepared. With a sharp razor blade, small pieces of *Pelargonium* plant material preferably from young leaves, or in few cases also from petals, and leaf pieces from internal standard were chopped up together in 500 μL of nuclei extraction buffer. After adding 1 mL staining solution plus 75 μL PI and 3 μL ribonuclease A, the nuclei suspension was gently shaken and afterwards filtered through a Cell-Strainer Cap (BD Falcon^™^) with a pore size of 35 μm. The measurements followed immediately after the sample preparation. At least three separate measurements were performed using the flow cytometer BD FACS Calibur^™^ (BD Biosciences) or CytoFLEX (Beckman Coulter). The separate measurements were secured by taking material from different plants of an accession or on different days. In cases of ambiguous peaks or a poor quality of the histogram peaks, additional measurements were performed (Table 1). Due to differences in the genome sizes and the occasional occurrence of endopolyploidy, it was sometimes difficult to assign the peaks correctly. In such cases, an overlapping of the peaks from the internal reference standard and *Pelargonium* could not be excluded. For this reason, measurements without reference standard were carried out followed by comparing the histograms in an overlay design to assign the peaks to corresponding origin. At least 5,000 events were recorded per measurement. For 2C DNA content the mean peak positions of internal reference standard and *Pelargonium* sample were analyzed supported by the analysis software BD CellQuest Pro (version 5.2.1) or CytExpert 2.3 (Beckman Coulter). The peak quality was assessed according to the CV value (coefficient of variance) and should always be below 5.0. If this was not the case, the measurement was repeated with newly chopped material. The nuclear DNA contents were calculated as proposed by Doležel *et al*. [39]:

Sample 2C value (DNA pg) = Reference 2C value x sample 2C mean peak position /reference 2C mean peak position. The 1C*x* DNA content was determined by dividing the 2C DNA content by the known ploidy.

### Statistical analysis

Data were analyzed by the statistical software Systat 13 (Germany) using, due to the different samples size of the accessions, the Tukey’s b test, α = 5%.

## Results

The 2C DNA content of 96 accessions from 60 *Pelargonium* species is presented (Table 1). *Pelargonium* is a genus with large differences in genome sizes between the species. The 2C DNA content in the genus ranges between 0.84 pg (*P*. *longifolium*) and 6.69 pg (*P*. *schizopetalum*) and per sections as follows: *Chorisma*: 2.53 pg—2.63 pg; *Jenkinsonia*: 2.37 pg—2.98 pg; *Myrrhidium*: 1.53 pg—2.16 pg; *Isopetalum*: 0.92 pg—0.95 pg; *Peristera*: 1.12 pg—2.57 pg; *Reniforma*: 1.60 pg—6.39 pg; *Ciconium*: 2.19 pg—4.54 pg; *Cortusina*: 1.06 pg—1.30 pg; *Hoarea*: 0.84 pg—1.77 pg; *Ligularia*: 1.40 pg—1.55 pg; *Magnistipulacea*: 2.89 pg—6.69 pg; *Otidia*: 1.30 pg—1.49 pg; *Pelargonium*: 0.95 pg—4.49 pg; *Polyactium*: 0.89 pg—4.29 pg, unassigned species: 3.45 pg—5.00 pg.

**Fig 1:**
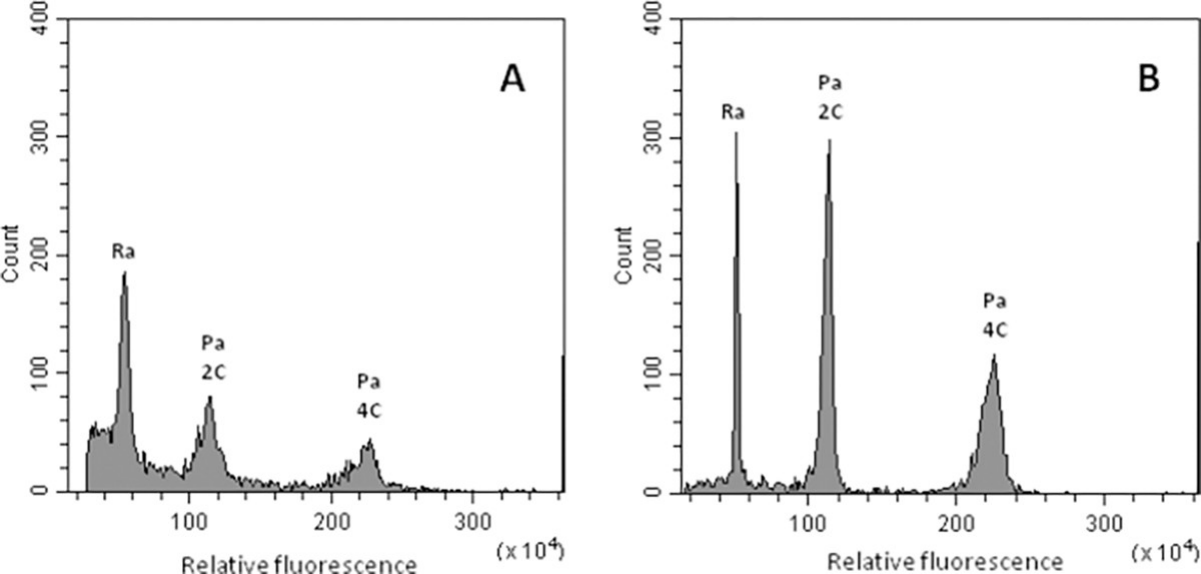
Fluorescence histograms of nuclei isolated from leaf tissue (A) and from petals (B) of *Pelargonium acetosum* accession 1/7. Ra: 2n = 2C peak of internal standard *Raphanus sativus*, Pa 2C / 4C: corresponding peaks of *P*. *acetosum*.

The quality of the measurements depends on the nuclei isolation procedure, the buffer and mainly on the quality of the plant material. In the present analysis, mostly leaf tissue was used because its easy accessibility. However, in some cases it turned to be difficult to obtain high quality histograms by using leaves as the tissue of choice. In such cases, petals were used as an alternative. As an example, Fig 1 shows representative histograms of nuclei of *P*. *acetosum* isolated either from leaf material (Fig 1A) or from petals (Fig 1B). Moreover, if leaf samples reveal a high degree of endopolyploidization making the interpretation of the histogram peaks difficult, the preparation of stained nuclei from petals can sometimes eliminate these problems. However, since the flowers were not always available, leaves were the most commonly used sample material. In addition to *P*. *acetosum*, also for *P*. *cortusifolium*, *P*. *peltatum*, and *P*. *sidoides* satisfying histograms were only obtain with nuclei isolated from petal tissue. In contrast, for *P*. *laxum* we could only clearly determine the 2C and the 4C DNA peaks in measurements of leaf tissue. Using petal tissue there was no 2C peak but a conspicuous 4C peak. Measurements with both, leaves and petals on 14 accessions of eight species have shown that the estimated genome size from petal samples was equal or slightly (but significantly) smaller and with one exception (*P*. *vitifolium* 39) significantly larger than from leaves samples (Table 2).

**Table 2:** Mean 2C DNA content (pg) of 14 *Pelargonium* accessions estimated with propidium iodide stained nuclei isolated from petals and leaves, respectively.

*Pelargonium* accession	FCM with petals	FCM with leaf material
	2C DNA content (pg)	2C DNA content (pg)
*P*. *acetosum* 1	2.31^a^ ± 0.06 (6)	2.40^b^ ± 0.00 (3)
*P*. *acetosum* 1/7	2.44^a^ ± 0.00 (3)	2.44^a^ ± 0.06 (5)
*P*. *acetosum* 102	2.43^a^ ± 0.02 (3)	2.54^a^ ± 0.05 (5)
*P*. *fulgidum* 11	1.50^a^ ± 0.04 (4)	1.58^b^ ± 0.02 (4)
*P*. *fulgidum* 48	1.51^a^ ± 0.03 (6)	1.58^b^ ± 0.01 (5)
*P*. *fulgidum* 123	1.48^a^ ± 0.02 (4)	1.52^b^ ± 0.01 (4)
*P*. *mollicomum* 131	2.52^a^ ± 0.11 (5)	2.57^a^ ± 0.11 (5)
*P*. *myrrhifolium* 22	1.54^a^ ± 0.04 (4)	1.59^b^ ± 0.05 (6)
*P*. *odoratissimum* 432	1.65^a^ ± 0.05 (6)	1.69^a^ ± 0.06 (4)
*P*. *peltatum* 26	2.19^a^ ± 0.02 (7)	2.24^b^ ± 0.05 (9)
*P*. *peltatum* 135	2.16^a^ ± 0.02 (10)	2.21^b^ ± 0.08 (12)
*P*. *peltatum* 506	2.19^a^ ± 0.06 (11)	2.20^b^ ± 0.03 (9)
*P*. *reniforme* 28	1.66^a^ ± 0.05 (4)	1.72^a^ ± 0.06 (5)
*P*. *vitifolium* 39	4.27^b^ ± 0.10 (5)	4.16^a^ ± 0.05 (2)

FCM: flow cytometry. Different letters in a line show significant differences, Tukey’s b test, α = 5%, the number of analysed samples per accession and tissue is indicated in parenthesis behind the standard deviation of 2C DNA content.

**Fig 2:**
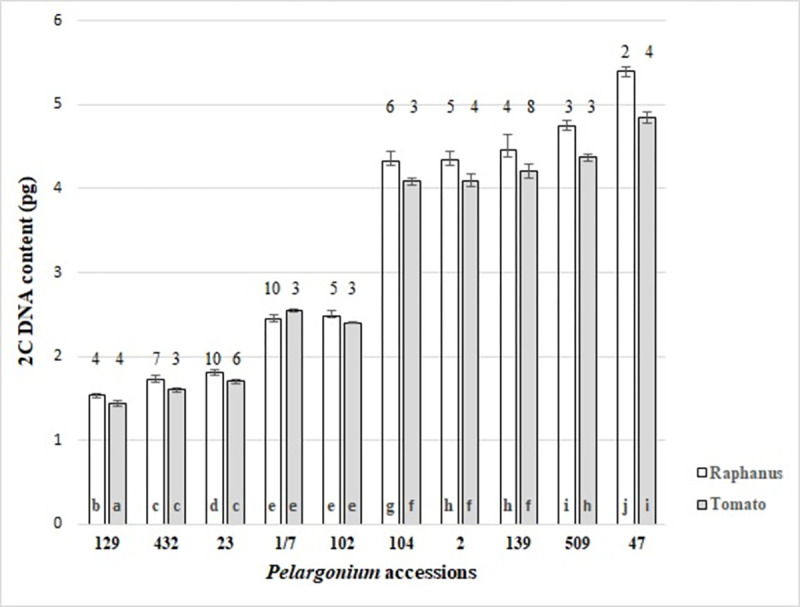
Comparison of flow cytometric 2C DNA content estimation of ten *Pelargonium* accessions using *Raphanus sativus* and tomato ‘Stupické’ as internal standard, different letters show significant differences, Tukey’s b test, α = 5%, numbers above the columns are numbers of the analyzed samples.

The genome sizes were determined using the two internal standards *R*. *sativus* or *S*. *lycopersicon*. As the only exception, *P*. *grandiflorum* was measured with cauliflower ‘Korso’ (Table 1). The choice of the standard depended on the position of the 2C sample peak at the x-axis of the histogram and on the CV values. The most important criterion for the selection of the standard was that its 2C DNA peak was close but sufficiently well separate from the 2C DNA *Pelargonium* peak. For instance, the estimation of the DNA content of *P*. *australe* (1.12 pg) and *P*. *echinatum* (1.09 pg) was only possible with tomato as internal standard due to an overlap of 2C DNA sample peak with the *R*. *sativus* 2C peak. Comparisons of measurements of ten accessions with both standards have shown that in seven accessions the estimated DNA content with tomato is significant lower than that with *R*. *sativus* (Fig 2). In three accessions (*P*. *odoratissmum* 432, *P*. *acetosum* 1/7 and 102) no significant differences between both internal standards were determined, although for *P*. *acetosum* 1/7 a higher DNA content was defined with the internal standard tomato.

**Fig 3:**
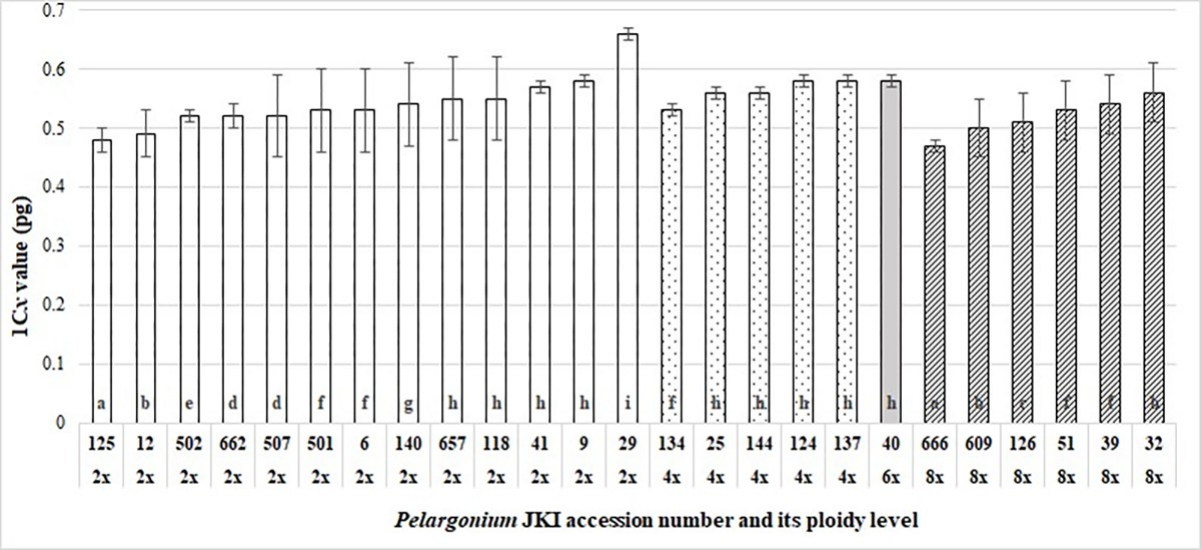
Mean 1C*x* value of 25 accessions of section *Pelargonium*. Different letters show significant differences, Tukey’s b test, α = 5%, n = 3–7.

Due to the breeders interests the *Pelargonium* JKI collection encompasses especially two sections: the section *Pelargonium* (25 accessions of 16 species) and *Ciconium* (26 accessions of 11 species). The 2C DNA content of the diploid species in this section amounted between 0.95 pg (*P*. *grandiflorum*) and 1.31 pg (*P*. *tabulare*), for the tetraploid ones between 2.13 pg and 2.32 pg, the hexaploid *P*. *capitatum* had 3.47 pg and the six 8*x* accessions ranged between 3.78 pg and 4.49 pg, respectively (Table 1). Within the section *Pelargonium*, a general relationship between the ploidy level of the accessions and their 1C*x* genome size was not found (Fig 3). The lowest 1C*x* DNA content was found for the octoploid accession *P*. *graveolens* 666 (0.47 pg) and the highest for the diploid *P*. *tabulare* 29 (0.66 pg) (Table 1).

Averaging the 1C*x* values over their ploidy level (Table 3), there is an increase from the diploid over the tetraploid species to the hexaploid species, but a significant decrease of mean 1C*x* value of the octoploid species compared to the mean 1C*x* values of the others ploidy levels (Table 3).

**Table 3:** Mean 1C*x* values (pg) of the accessions of the section *Pelargonium* according to the different ploidy levels.

Ploidy level	№ of accessions	№ of samples	Mean 1C*x* value (pg)	SD
2*x*	13	49	0.54^b^	0.04
4*x*	5	20	0.56^b^	0.02
6*x*	1	3	0.58^b^	0.01
8*x*	6	28	0.52^a^	0.03

Different letters in the column show significant differences, Tukey’s b test, α = 5%, SD: standard deviation.

The section *Ciconium* includes the species *P*. *zonale* and *P*. *peltatum* that have so far mostly been used horticulturally. Therefore, this section is of high interest as genetic resource for breeding efforts. We estimated the genome size of 26 accessions of 11 *Ciconium* species at two ploidy levels, 2*x* and 4*x*. For some accessions, the ploidy is not yet clear. Among the diploid accessions, *P*. *peltatum* has the smallest genome (2.19 pg) whereas *P*. *tongaense* has the largest one with 2.77 pg. At the tetraploid level, a 2C DNA content from 3.57 pg (*P*. *multibracteatum*) to 4.55 pg (*P*. *zonale*) was determined. Although *P*. *quinquelobatum* was described as diploid our measurement of 2C = 4.54 pg indicates that the *P*. *quinquelobatum* accession of the JKI collection is tetraploid.

The two tetraploid *P*. *multibracteatum* accessions show a noticeable small 1C*x* value of 0.89 and 0.90 pg, while 1C*x* values of the other accessions of this section are between 1.10 pg (*P*. *peltatum*, 2*x*) and 1.25 pg (*P*. *acraeum*, 2*x*). *Pelargonium tongaense* (2*x*) has a strikingly higher 1C*x* DNA content of 1.38 pg. Averaging the 1C*x* values over the respective ploidy level, the diploid mean 1C*x* value (1.15 pg, 20 accessions, 153 samples) is significant higher than tetraploid mean 1C*x* value (1.02 pg, 6 accessions, 42 samples). Within the subgenus *Paucisignata*, which includes the section *Ciconium* and two unassigned species, one accession of *P*. *caylae* revealed the highest 2C DNA content (5.00 pg) and *P*. *transvaalense* the highest 1C*x* DNA content (1.72 pg), respectively.

Summarizing the 1C*x* DNA contents according to the section and ploidy level (Table 4), the diploid and tetraploid accession of the section *Hoarea* (0.42 pg and 0.44 pg) and the diploid accession of the section *Polyactium* (0.44 pg) show the lowest values. Highest 1C*x* DNA contents (1.32 pg and 1.33 pg) of the genus *Pelargonium* were estimated for the sections *Chorisma* and *Jenkinsonia*. The mean 1C*x* DNA content of the genus *Pelargonium* is 0.89 pg, including unassigned species (Table 1), which are not integrated in Table 4.

**Table 4:** Summary of the 1C*x* DNA contents of the analyzed *Pelargonium* sections regarding chromosome size, basic chromosome number and ploidy level.

Section	№ Accessions	Chromosome size (μm)	№ of chromo-somes (n)	Ploidy level (*x*)	1C*x* DNA content (pg)
*Hoarea*	1	< 1.5	11	2	0.42
*Hoarea*	1	< 1.5	11	4	0.44
*Polyactium*	1	< 1.5	11	2	0.44
*Isopetalum*	2	< 1.5	8	2	0.47
*Pelargonium*	6	< 1.5	11	8	0.52
*Pelargonium*	13	< 1.5	11	2	0.54
*Peristera*	1	< 1.5	9	2	0.56
*Pelargonium*	5	< 1.5	11	4	0.56
*Cortusina*	5	< 1.5	11	2	0.58
*Pelargonium*	1	< 1.5	11	6	0.58
*Peristera*	1	< 1.5	8, 19	4	0.64
*Otidia*	4	< 1.5	11	2	0.70
*Polyactium*	2	< 1.5	11	6	0.71
*Magnistipulacea*	1	< 1.5	11	4	0.72
*Ligularia*	4	< 1.5	11	2	0.76
*Reniformia*	1	< 1.5	8	8	0.80
*Reniformia*	4	< 1.5	8	2	0.84
*Reniformia*	2	< 1.5	8	4	0.86
*Myrrhidium*	3	> 1.5	11	2	0.90
*Ciconium*	6	> 1.5	9	4	1.02
*Ciconium*	20	> 1.5	9	2	1.15
*Chorisma*	3	> 1.5	11	2	1.32
*Jenkinsonia*	3	> 1.5	9	2	1.33

Independent of the basic chromosome number, sections with a chromosome size < 1.5 μm possess lower 1C*x* DNA contents than sections with a chromosome size >1.5 μm. The sections *Reniforma* (< 1.5 μm; 0.84 pg and 0.86 pg) and *Myrrhidum* (>1.5 μm; 0.90 pg) mark apparently the transition regarding chromosome and genome size. Considering ploidy levels, 1C*x* DNA contents increase until *6x* (*Hoarea*, *Pelargonium*, *Peristera*, *Reniforma*), but decrease at *8x* (*Pelargonium*, *Reniforma*). As described above, section *Ciconium* is an exception.

Of the other twelve sections, only two to five species were examined. Despite the small sample size, the high plasticity of the *Pelargonium* genomes is demonstrated. Especially the representatives of section *Reniformia* reveal a variable genome size from 1.60 pg for *P*. *ionidiflorum* to 6.39 pg for *P*. *sidoides*. Obviously, different levels of ploidy exist between and even within the species. In section *Magnistipulacea*, the 2C DNA content for tetraploid *P*. *bowkeri* amounted 2.89 pg and for *P*. *schizopetalum* to 6.69 pg. For the latter one, the chromosome number is not yet determined. The three examined accessions of the sections *Chorisma* and *Jenksonia* have a similar genome size between 2.37 and 2.91 pg. In section *Myrrhidium* three varieties of *P*. *myrrhifolium* were tested. Two of them have a 2C DNA content of 1.55 and 1.53 pg, respectively. With 2.16 pg *P*. *myrrhifolium* var. *myrrhifolium* differs significantly from the other two varieties.

To investigate the intraspecific variability of 2C DNA content, nine species with three or more accessions were statistically analyzed [S2 Table]. The accessions of the species *P*. *peltatum*, *P*. *caylae*, and *P*. *fulgidum* show no significant intraspecific differences of the 2C DNA content, whereas for the accessions of *P*. *acetosum*, *P*. *cucullatum*, *P*. *graveolens*, *P*. *myrrhifolium*, and *P*. *vitifolium* a significant intraspecific variability was found. Regarding *P*. *zonale*, the five diploid accessions do not differ significantly. After chromosome doubling by colchicine treatment the *P*. *zonale* accession 509 is tetraploid. This was confirmed again.

## Discussion

Species of genus *Pelargonium* are of interest for both, botanists and ornamental plant producers. In the last century, cytological investigations revealed variability in chromosome number and size [12, 14, 15, 40–42]. With the implementation of molecular methods, *Pelargonium* phylogenetic relationships were more deeply investigated [5, 7, 19, 22–25, 43, 44]. According to new molecular insights, some changes in the phylogenetic systematics of the genus *Pelargonium* have been proposed [2, 45].

So far, the genome size has been determined for many plants species but information about *Pelargonium* is limited. In the publicly accessible Plant DNA C-values Database [46] 2C DNA values are only listed for *P*. *radula* (16.20 pg) [31] and 28 other *Pelargonium* species determined by Weng *et al*. [22]. All of the genome sizes determined by us differ considerably from the values given by Greilhuber [31] and Weng *et al*. [22], respectively. The largest genome determined by us is that for *P*. *schizopetalum* with 6.69 pg (2C). Greilhuber [31] determined the DNA amount by Feulgen method. *Pelargonium radula* is a synonym for *P*. *radens* H.E. Moore [1] belonging to section *Pelargonium* [12]. We examined the octoploid species *P*. *graveolens* and *P*. *vitifolium* of the same section and found DNA amounts between 3.78 and 4.49 pg. This was much lower than the DNA amount of 16.20 pg for *P*. *radula* found by Greilhuber [31]. Since we observed that the 1C*x* content of species belonging to the section *Pelargonium* varies only a little, the large genome of *P*. *radula* is rather surprising. However, since *P*. *radula* was not included in our measurements and the chromosome number of the horticultural accession investigated by Greilhuber is given with 2n = 80–82 the reasons for this variation remains elucidated.

With the exception of *P*. *tetragonum*, the DNA values determined by Weng *et al*. [22], which are also published in The Plant DNA C-values Database (Royal Botanic Gardens, Kew), are always much smaller than the DNA values presented here. We can only speculate about the reasons for the substantial deviation from our results. According to Weng *et al*. [22], the samples were chopped and stained with PI. *Arabidopsis thaliana* (L.) Heynh. or pre-stained control trout (*Oncorhynchus mykiss* Walbaum) erythrocytes (DNA control PI #05–7303, Partec^®^, Germany) served as internal standards. The separately stained samples and standards were mixed together immediately before measurement. This type of standardization harbours errors, as the sample and standard were prepared in different environments. The average of the two independent estimates is reported as 2C DNA value. The authors have not given the individual values with each internal standard. Therefore, it is difficult to find the reason for the significant differences from our results. However, we can state at least that both standards are not the best choice. *Arabidopsis* has a very small DNA amount and often reveals an extensive endopolyploidy [47]. Hence, *Arabidopsis* is not recommended as reference standard due to a potential misinterpretation of the origin of histogram peaks. The second standard, the pre-stained control trout erythrocytes, is biologically far away from plant cells that may contain staining inducing substances in its cytosol. Therefore, animal standards are not recommended for plant samples [39, 48]. Moreover, Partec^®^, now Sysmex^®^, Germany, the producer of the DNA control PI # 05–7303 advises it as control for the instruments linearity but not for a DNA content determination.

Another comprehensive study on genome size in *Pelargonium* was published by Nieuwenhuis [34], who collected samples from two botanical gardens. As internal standard *Vinca minor* L. (2C DNA value = 1.51 pg) was used. Since most of the samples were measured with DAPI, Nieuwenhuis comparatively analysed 14 accessions with DAPI and PI to introduce a conversion factor that allows to correcting the obtained DAPI values for differences in AT-CG base pair portions. Our results are in good concordance with the DNA amounts estimated by Nieuwenhuis [34]. However, large differences were found for few species. Moreover, for many species several cytotypes were reported. From this, we conclude, for example, that our accession *P*. *alchemilloides* (4.24 pg) is tetraploid whereas Nieuwenhuis [34] determined a diploid *P*. *alchemilloides* (2.15 pg). Some results of the description of *Pelargonium* JKI collection require further clarification regarding the botanical classification. By handing over the accessions over many years and many hands, errors cannot be ruled out. Furthermore, we cannot exclude breeding efforts such as polyploidization. To our knowledge, this is the first report about a diploid *P*. *crithmifolium* (1.37 pg), tetraploid *P*. *quinquelobatum* (4.54 pg) and *P*. *oblongatum* (1.77 pg) as well as an octoploid *P*. *sidoides* (6.39 pg).

Endopolyploidy in plants is a common phenomenon [49]. Few *Pelargonium* accessions revealed a high degree of endopolyploidy in the leaves. In such cases, it is difficult to avoid misinterpretations of the histogram [50]. Barow and Meister [51] have shown that the degree of endopolyploidization differs between the different organs of a given species and between the different life-cycle types. We repeated measurements at different times throughout the year, have taken leaves at different ages (lower or upper leaves) or, in addition to the leaf material, we also used petals for the measurement. Only in this way, it was possible to determine the 2C peak in *P*. *peltatum*, *P*. *acetosum*, and *P*. *laxum*.

Beside a different histogram quality, DNA contents from petals are lower as DNA contents defined with leaf material or do not differ, with only one exception, namely *P*. *vitifolium*. In contrast to our three investigated *P*. *fulgidum* accessions, where the DNA content of petal samples was lower, Nieuwenhuis’ [34] analysis of leaf and petal samples of one *P*. *fulgidum* accession resulted in no significant differences of the DNA content. Furthermore, comparing the applied standards, we have found out that the determined DNA content with internal reference standard *R*. *sativus* was equal to or higher than the genome size determined with *S*. *lycopersicon*. Therefore, both, the type of tissue and the used internal standard, may influence the DNA content estimation. Greilhuber *et al*. [48] have already discussed methodological aspects of preparing samples for DNA content measurements with special consideration of standardization and the role of different cytosol compounds as fluorescence inhibitors. Up to now chemical identities of influencing substances from the cytosol are poorly explored [50]. An additional effect could have small particles e.g. coming from trichomes or other parts of the chopped plant tissue. The debris could aggregate with the stained nuclei and can lead to an apparent increase in nuclear fluorescence [52]. Even for a skilled person in the laboratory it is difficult to chop exactly the same amounts from target and standard tissue for preparing the sample. Furthermore, a high amount of extracted secondary metabolites [53] or simply a hidden infestation of the plants with whiteflies [54] could affect the results adversely. Taken these facts altogether it makes standardization between different laboratories difficult or almost impossible and plant 2C DNA contents even for the same species could differ in a small tolerance range. Despite these general drawbacks, flow cytometry is an acknowledged way to determine the genome size of plant species. The advantages over cytological examinations such as simplicity and speed have often been described [32]. Additionally, if the basic chromosome number is known, then the estimation of the 1C*x* value is a further valued feature of species. For example, a 1C*x* value downsizing was often reported after polyploidization [35, 55]. Nieuwenhuis [34] described for the genus *Pelargonium* a decrease of 1C*x* values with increasing ploidy levels during evolution. The extensively examined section *Pelargonium* with four ploidy levels shows an averaged genome upsizing from the diploid over the tetraploid to the hexaploid species, but a significant genome downsizing of the octoploid species compared to the other ploidy levels. Regarding our results, a general conclusion, if evolutionary or induced polyploidization leads to a genome upsizing or downsizing in the genus *Pelargonium*, is impossible and further investigations are necessary.

As expected, the chromosome size correlates with the 1C*x* value. Species with small chromosomes have a remarkable lower 1C*x* value than species with larger chromosomes regardless the basic chromosome number. Interestingly, the three examined accessions of the sections *Chorisma* and *Jenkinsonia*, have a similar genome size between 2.37 and 2.91 pg despite similar chromosome size and different basic chromosome number, namely 11 in *Chorisma* and 9 in *Jenkinsonia* [15, 56–58]. In *P*. *sidoides*, *P*. *quinquelobatum*, *P*. *oblongatum*, and *P*. *crithmifolium* the result deviates strongly from the expected 1C*x* value. One possible explanation is that the accessions possess a ploidy that has not yet been described. Intraspecific variability in genome size could be explained by the different provenance of the accessions, the existence of subspecies (*P*. *cucullatum* [59, 60], *P*. *myrrhifolium* [61]) or different cytotypes, but also by diverse breeding efforts as induced polyploidization e.g. for *P*. *zonale*. Additionally, the here presented and already published 2C and 1C*x* DNA contents of the genus *Pelargonium* [22, 34] are summarized in S3 Table.

In summary, it could be concluded that plant flow cytometry is a powerful tool for characterization of genetic resources in the genus *Pelargonium*. For the *Pelargonium* JKI collection, the flow cytometric data are basics for the plant accession characterization. The data presented here encompass 559 measurements under different conditions with internal standards. Additionally, numerous measurements were performed without internal reference standard for clarifying of the sample 2C and 4C peak position on the histogram. The DNA content was determined for 60 *Pelargonium* species of it for 22 *Pelargonium* species for the first time. The reported genome sizes give interesting insights in the accessions of the *Pelargonium* JKI collection and serve, together with the morphological traits, as a basic passport for the accessions. Furthermore, they are valuable for future *Pelargonium* genome sequencing programs.

## Supporting information

S1 Table Full scientific names of *Pelargonium* species / accessions. (DOCX)

S2 Table Analysis of intraspecific genome size variation in nine *Pelargonium* species. (DOCX)

S3 Table Summary of available 2C and 1C*x* DNA contents of *Pelargonium* species reported in this study, by Nieuwenhuis [34] and Weng *et al*. [22]. (DOCX)

## References

[1] Van der Walt JJA. Pelargoniums of southern Africa. Vol. 1, 2nd ed. Hillscheid: Fischer; 1979.

[2] Röschenbleck J, Albers F, Müller K, Weinl S, Kudla J. Phylogenetics, character evolution and a subgeneric revision of the genus *Pelargonium* (Geraniaceae). Phytotaxa. 2014; 159: 31–76. 10.11646/phytotaxa.159.2.1

[3] Albers F, Gibby M, Austmann M. A reappraisal of *Pelargonium* sect. *Ligularia* (*Geraniaceae*). Pl Syst Evol. 1992; 179: 257–276. 10.1007/BF00937601.

[4] Potts AJ, Hedderson TA, Vlok JHJ, Cowling RM. Pleistocene range dynamics in the eastern Greater Cape Floristic Region: A case study of the Little Karoo endemic *Berkheya cuneate* (Asteraceae). S Afr J Bot. 2013; 88: 401–413. 10.1016/j.sajb.2013.08.009.

[5] Bakker FT, Culham A, Marais EM, Gibby M. Nested radiation in Cape *Pelargonium*. In: Bakker FT, Chartrou LW, editors. Plant species-level systematics: New perspectives on pattern and process. Koenigstein: Koeltz Scientific Books; 2005. pp. 75–100.

[6] Verboom GA, Archibald JK, Bakker FT, Bellstedt DU, Conrad F, Dreyer LL et al. Origin and diversification of the Greater Cape flora: ancient species repository, hot-bed of recent radiation, or both? Mol Phylogenet Evol. 2009; 51, 44–53. doi: 10.1016/j.ympev.2008.01.037 18411064

[7] Van de Kerke SJ, Shrestha B, Ruhlman TA, Weng ML, Jansen RK, Jones CS et al. Plastome based phylogenetics and younger crown node age in *Pelargonium*. Mol Phylogenet Evol. 2019; 137: 33–43. doi: 10.1016/j.ympev.2019.03.021 30926482

[8] Bakker FT, Gibby M, Culham A. Phylogenetics and diversification in Pelargonium. In: Hollingsworth PM, Bateman R, Gornall RJ, editors. Molecular Systematics and Plant Evolution. London: Chapman & Hall; 1999. pp. 353–374. 10.1201/9781439833278.ch16.

[9] Becker M, Schäper K, Albers F. Description of two new taxa of *Pelargonium* section *Otidia* (Geraniaceae), *P*. *keeromsbergense* and *P*. *laxum* ssp. *karooicum*. Schumannia. 2008; 5: 181–190.

[10] Manning JC, le Roux A. *Pelargonium conradiae* (Geraniaceae), a new species in section *Ligularia* from Worcester, Western Cape, South Africa. S Afr J Bot. 2016; 105: 313–316. 10.1016/j.sajb.2016.03.014.

[11] Marais EM. Five new species of *Pelargonium*, section *Hoarea* (Geraniaceae), from the Western and Northern Cape Provinces of South Africa. S Afr J Bot. 2016; 103: 145–155. 10.1016/j.sajb.2015.09.007.

[12] Albers F, van der Walt JJA. Untersuchungen zur Karyologie und Mikrosporogenese von *Pelargonium* sect. *Pelargonium (Geraniaceae)*. Pl Syst Evol. 1984; 147: 177–188. 10.1007/BF00989382.

[13] Gibby M, Westfold J. A new basic chromosome number in *Pelargonium* (Geraniaceae). Caryologia. 1983; 36: 79–82. 10.1080/00087114.1983.10797646.

[14] Gibby M, Westfold J. A cytological study of *Pelargonium* sect. *Eumorpha (Geraniaceae)*. Plant Syst Evol. 1986; 153: 205–222. 10.1007/BF00983688.

[15] Gibby M, Albers F, Prinsloo B. Karyological studies in *Pelargonium* sectt. *Ciconium*, *Dibrachya*, and *Jenkinsonia (Geraniaceae)*. Plant Syst Evol. 1990; 170: 151–159. 10.1007/BF00937700.

[16] Renou J-P, Aubry C, Serveau M, Jalouzot P. Evaluation of the genetic variability in the genus *Pelargonium* using RAPD markers. J Hort Sci. 1997; 72: 229–237. 10.1080/14620316.1997.11515510.

[17] Bakker FT, Culham A, Pankhurst CE, Gibby M. Mitochondrial and chloroplast DNA-based phylogeny of *Pelargonium* (Geraniaceae). Am J Bot. 2000; 87: 727–734. 10.2307/2656859. 10811797

[18] Plaschil S, Budahn H, Wiedemann M, Olbricht K. Genetic characterization of *Pelargonium* L’Hér. germplasm. Genet Resour Crop Evol. 2017; 64: 1051–1059. 10.1007/s10722-016-0424-x.

[19] Bakker FT, Culham A, Hettiarachi P, Touloumenidou T, Gibby M. Phylogeny of Pelargonium (Geraniaceae) based on DNA sequences from three genomes. Taxon. 2004; 53: 17–28. 10.2307/4135485.

[20] Bakker FT, Breman F, Merckx V. DNA sequence evolution in fast evolving mitochondrial DNA nad1 exons in Geraniaceae and Plantaginaceae. Taxon. 2006; 55: 887–896. 10.2307/25065683.

[21] Guisinger MM, Kuehl JV, Boore JL, Jansen RK. Genome-wide analyses of Geraniaceae plastid DNA reveal unprecedented patterns of increased nucleotide substitutions. PNAS. 2008; 105: 18424–18429. doi: 10.1073/pnas.0806759105 19011103 PMC2587588

[22] Weng ML, Ruhlman TA, Gibby M, Jansen RK. Phylogeny, rate variation, and genome size evolution of *Pelargonium* (Geraniaceae). Mol Phylogenet Evol. 2012; 64: 654–670. doi: 10.1016/j.ympev.2012.05.026 22677167

[23] Weng ML, Blazier JC, Govindu M, Jansen RK. Reconstruction of the ancestral plastid genome in Geraniaceae reveals a correlation between genome rearrangements, repeats, and nucleotide substitution rates. Mol Biol Evol. 2013; 31: 645–659. doi: 10.1093/molbev/mst257 24336877

[24] Weng ML, Ruhlman TA, Jansen RK. Plastid-nuclear interaction and accelerated coevolution in plastid ribosomal genes in Geraniaceae. Genome Biol Evol. 2016; 8: 1824–1838. doi: 10.1093/gbe/evw115 27190001 PMC4943186

[25] Weng ML, Ruhlman TA, Jansen. Expansion of inverted repeat does not decrease substitution rates in *Pelargonium* plastid genomes. New Phytol. 2017; 214: 842–851. doi: 10.1111/nph.14375 27991660

[26] Apitz J, Weihe A, Pohlheim F, Börner T. Biparental inheritance of organelles in *Pelargonium*: evidence for intergenomic recombination of mitochondrial DNA. Planta. 2013; 237: 509–515. doi: 10.1007/s00425-012-1768-x 23053540

[27] Röschenbleck J, Wicke S, Weinl S, Kudla J, Müller KF. Genus-wide screening reveals four distict types of structural plastid genome organization in *Pelargonium* (Geraniaceae). Genome Bio Evol. 2017; 9: 64–70. doi: 10.1093/gbe/evw271 . 28172771 PMC5381562

[28] Choi KS, Weng ML, Ruhlman TA, Jansen RK. Extensive variation in nucleotide substitution rate and gene/intron loss in mitochondrial genomes of *Pelargonium*. Mol Phylogenet and Evol. 2021; 155: 106986. doi: 10.1016/j.ympev.2020.106986 33059063

[29] Breman, FC. Exploring patterns of cytonuclear incompatibility in *Pelargonium* section *Ciconium*. PhD Thesis, Wageningen University. 2021. Available from: https://www.researchgate.net/publication/354598481_Exploring_patterns_of_cytonuclear_incompatibility_in_Pelargonium_section_Ciconium. 10.18174/551565.

[30] Federal Plant Variety Office, Germany. [cited 2022 Jan 13]; Available from: https://www.bundessortenamt.de/bsa/en/plant-genetic-resources/german-gene-bank-for-ornamentals/german-gene-bank-for-vegetatively-propagated-ornamentals.

[31] Greilhuber J. “Self-tanning” a new and important source of stoichiometric error in cytophotometric determination of nuclear DNA content in plants. Plant Syst and Evol. 1988; 158: 87–96. 10.1007/BF00936335.

[32] Sliwinska E. Flow cytometry–a modern method for exploring genome size and nuclear DNA synthesis in horticultural and medicinal plant species. Folia Horticulturae. 2018; 30: 103–128. 10.2478/fhort-2018-0011.

[33] Cassells AC, Croke JT, Doyle BM. Evaluation of image analysis, flow cytometry, and RAPD analysis for the assessment of somaclonal variation and induced mutation in tissue culture derived *Pelargonium* plants. J Appl Bot. 1997; 71: 125–130.

[34] Nieuwenhuis M. Evolutionary trends in genome size and polyploidy in Pelargonium (Geraniaceae). M.Sc. Thesis, Wageningen University & Research. 2013. Available from: https://www.researchgate.net/publication/335259912_Evolutionary_trends_in_genome_size_and_polyploidy_in_Pelargonium_Geraniaceae?channel=doi&linkId=5d5bb554458515210252446f&showFulltext=true#fullTextFileContent. 10.13140/RG.2.2.25087.36005.

[35] Leitch IJ, Bennett MD. Genome downsizing in polyploid plants. Biol J Linn Soc. 2004; 82: 651–663. doi:10.1111/j.1095-8312.2004.00349.x.

[36] Doležel J, Sgorbati S, Lucretti S. Comparison of three DNA fluorochromes for flow cytometric estimation of nuclear DNA content in plants. Physiol Plant. 1992; 85: 625–631. 10.1111/j.1399-3054.1992.tb04764.x.

[37] Plaschil S, Abel S, Klocke E. Flow cytometric investigations on *Pelargonium* × *crispum*: an estimation of nuclear DNA contents with two different internal standards. J Kulturpflanzen. 2020; 72: 236–242. 10.5073/JfK.2020.06.04.

[38] Murashige T, Skoog F. 1962. A revised medium for rapid growth and bioassays with tobacco tissue cultures. Physiol Plant. 1962; 15: 473–497. 10.1111/j.1399-3054.1962.tb08052.x.

[39] Doležel J, Greilhuber J, Suda J. Estimation of nuclear DNA content in plants using flow cytometry. Nat Protoc. 2007; 2: 2233–2244. doi: 10.1038/nprot.2007.310 17853881

[40] Daker MG. Chromosome number of *Pelargonium* species and cultivars. J R Horti Soc. 1969; 94: 346–353.

[41] Yu S-N, Horn WAH. Additional chromosome numbers in *Pelargonium* (*Geraniaceae*). Plant Syst Evol. 1988; 159: 165–171. 10.1007/BF00935969.

[42] Gibby M, Hinnah S, Marais EM, Albers F. Cytological variation and evolution within *Pelargonium* section *Hoarea (Geraniaceae)*. Plant Syst Evol. 1996; 203: 111–142. 10.1007/BF00985241.

[43] Touloumenidou T, Bakker FT, Marais EM et al. Chromosomal evolution interpreted from the rDNA ITS phylogeny for *Pelargonium* sect. *Hoarea* (Geraniaceae). Schumannia. 2004; 4: 93–106.

[44] Becker M, Albers F. Taxonomy and phylogeny of two subgroups of *Pelargonium* section *Otidia* (Geraniaceae). 1. The *Pelargonium carnosum* complex. Bothalia. 2009; 39: 73–85. 10.4102/abc.v39i1.231.

[45] Albers F, van der Walt JJA, Gibby M, Marschewski DE, van der Merwe AM, Marais EM, et al. Biosystematic study of *Pelargonium* section *Ligularia*: 4. The section *Ligularia sensu stricto*. S Afr J Bot. 2000; 66: 31–43. 10.1016/S0254-6299(15)31049-8.

[46] Pellicer J, Leitch IJ. 2019. The Plant DNA C-values Database (release 7.1): an updated online repository of plant genome size data for comparative studies. The Physiologist. 5 pp. doi: 10.1111/nph.16261 31608445

[47] Galbraith DW, Harkins KR, Knapp S. Systemic endopolyploidy in *Arabidopsis thaliana*. Plant Physiol. 1991; 96: 985–989. doi: 10.1104/pp.96.3.985 . 16668285 PMC1080875

[48] Greilhuber J, Temsch EM, Loureiro JCM. Nuclear DNA content measurement. In: Doležel J Greilhuber J, Suda J, editors. Flow cytometry with plant cells. Weinheim: Wiley-VCH; 2007. pp. 67–101.

[49] Leitch IJ, Dodsworth S. Endopolypoidy in plants. In: eLS. Chichester: John Wiley & Sons, Ltd; 2017. pp. 1–10. 10.1002/9780470015902.a0020097.pub2.

[50] Pellicer J, Powell RF, Leitch IJ. The application of flow cytometry for estimating genome size, ploidy level endopolyploidy, and reproductive modes in plants. Methods Mol Biol. 2021; 2222: 325–361. doi: 10.1007/978-1-0716-0997-2_17 33301101

[51] Barow M, Meister A. Endopolyploidy in seed plants is differently correlated to systematics, organ, life strategy and genome size. Plant Cell Environ. 2003; 26: 571–584. 10.1046/j.1365-3040.2003.00988.x.

[52] Loureiro J, Rodriguez E, Doležel J, Santos C. Flow cytometric and microscopic analysis of the effect of tannic acid on plant nuclei and estimation of DNA Content. Ann Bot. 2006; 98: 515–527. doi: 10.1093/aob/mcl140 . 16820406 PMC2803573

[53] Doležel J, Bartoš J. Plant DNA Flow Cytometry and Estimation of Nuclear Genome Size: Ann Bot. 2005; 95: 99–110. doi: 10.1093/aob/mci005 . 15596459 PMC4246710

[54] Brown JK, Lambert GM, Ghanim M, Czosnek H, Galbraith DW. Nuclear DNA content of the whitefly *Bemisia tabaci* (Aleyrodidae: Hemiptera) estimated by flow cytometry. Bull Entomol Res. 2005; 95: 309–312. doi: 10.1079/ber2005361 16048678

[55] Zenil-Ferguson R, Ponciano JM, Burleigh JG. Evaluating the role of genome downsizing and size thresholds from genome size distributions in angiosperms. Am J Bot. 2016; 103: 1175–1186. doi: 10.3732/ajb.1500408 27206462

[56] Scheltema AG & van der Walt JJA. Taxonomic revision of *Pelargonium* section *Jenkinsonia* (Geraniaceae) in southern Africa. S Afr J Bot. 1990; 56: 285–302. 10.1016/S0254-6299(16)31056-0.

[57] Albers F, van der Walt JJA, Gibby M, Marschewski D. A biosystematic study of *Pelargonium* section *Ligularia*: 2. Reappraisal of section *Chorisma*. S Afr J Bot. 1995; 61: 339–346. 10.1016/S0254-6299(15)30556.

[58] Van der Walt JJA, Albers F, Gibby M, Marschewski DE, Hellbrügge D, Price RA, et al. A biosystematic study of *Pelargonium* section *Ligularia*: 3. Reappraisal of section *Jenkinsonia*. S Afr J Bot. 1997; 63: 4–21. 10.1016/S0254-6299(15)30686-4.

[59] Volschenk B, van der Walt JJA, Vorster PJ. The subspecies of *Pelargonium cucullatum* (Geraniaceae). Bothalia. 2009; 14: 45–51. 10.4102/abc.v14i1.1134.

[60] Van der Walt JJA. A taxonomic revision of the type section *Pelargonium* L’Herit. (Geraniaceae). Bothalia. 1985; 15: 345–385. 10.4102/abc.v15i3/4.1828.

[61] Van der Walt JJA, Boucher DA. 1985. A taxonomic revision of the section *Myrrhidium* of *Pelargonium* (Geraniaceae) in southern Africa. S Afr J Bot. 52: 438–462. 10.1016/s0254-6299(16)31508-3.

