# Supplementary material for: The variability of nuclear DNA content of different Pelargonium species estimated by flow cytometry
Source: PLoS One. 2022 Apr 28;17(4):e0267496. doi: 10.1371/journal.pone.0267496 (PMC9049363; doi:10.1371/journal.pone.0267496)
Supplement: S1 Table — (DOCX) [file pone.0267496.s001.docx]

**S1 Table. Full scientific names of *Pelargonium* species / accessions according to Röschenbleck *et al.* (2014), with JKI collection number as well as number of Database of German Gene Bank for Ornamentals (DGZ).**

| **Subgenus** | **Species** | **№ JKI Accession** | **№ Database German Gene Bank for Ornamentals*** |
| --- | --- | --- | --- |
| ***Magnipetala*** | Section ***Chorisma* (Lindley ex Sweet) DC.** |  |  |
|  | *P. mollicomum* Fourc. | 131 | DEU648PELAR054 |
|  | *P. tetragonum* (L. f.) L'Hér. | 30 | DEU648PELAR079 |
|  | *P. worcesterae* R. Knuth | 72 | DEU648PELAR088 |
|  |  |  |  |
|  | Section ***Jenkinsonia* (Sweet) DC.** |  |  |
|  | *P. mutans* Vorster | 133 | DEU648PELAR057 |
|  | *P. mutans* Vorster | 316 | n.d. |
|  | *P. trifidum* Jacq. | 147 | DEU648PELAR084 |
|  |  |  |  |
|  | Section ***Myrrhidium* DC.** |  |  |
|  | *P. myrrhifolium* (L.) L'Hér. var. *myrrhifolium* | 20 | DEU648PELAR058 |
|  | *P. myrrhifolium* var. *coriandrifolium* (L.) Harv. | 22 | DEU648PELAR060 |
|  | *P. myrrhifolium* (var. *synnotii*) | 21 | DEU648PELAR059 |
|  |  |  |  |
| ***Parvulipetala*** | Section ***Isopetalum* (Sweet) DC.** |  |  |
|  | *P. cotyledonis* (L.) L'Hér. | 74 | DEU648PELAR021 |
|  | *P. cotyledonis* (L.) L'Hér. | 116 | DEU648PELAR022 |
|  |  |  |  |
|  | Section ***Peristera* DC.** |  |  |
|  | *P. australe* Willd. | 109 | DEU648PELAR010 |
|  | *P. grossularioides* (L.) L'Hér. | 13 | DEU648PELAR043 |
|  | *P. rodneyanum* T. Mitch. ex Lindl. | 65 | n.d. |
|  |  |  |  |
|  | Section ***Reniformia* (R. Knuth) Dreyer** |  |  |
|  | *P. abrotanifolium* (L. f.) Jacq. | 101 | DEU648PELAR001 |
|  | *P. ionidiflorum* (Eckl. & Zeyh.) Steud. | 73 | DEU648PELAR048 |
|  | *P. odoratissimum* (L.) L'Hér. | 52 | DEU648PELAR062 |
|  | *P. odoratissimum* (L.) L'Hér. | 432 | DEU648PELAR063 |
|  | *P. reniforme* (Andrews) Curtis subsp. *reniforme* | 28 | DEU648PELAR074 |
|  | *P. sidoides* DC. | 142 | DEU648PELAR077 |
|  | *P. sidoides* DC. | 321 | n.d. |
|  |  |  |  |
| ***Paucisignata*** | Section ***Ciconium* (Sweet) Harv.** |  |  |
|  | *P. acetosum* (L.) L'Hér. | 1 | DEU648PELAR002 |
|  | *P. acetosum* (L.) L'Hér. | 1/7 | n.d. |
|  | *P. acetosum* (L.) L'Hér. | 102 | DEU648PELAR003 |
|  | *P. acraeum* R.A. Dyer | 103 | DEU648PELAR004 |
|  | *P. alchemilloides* (L.) L'Hér. | 2 | DEU648PELAR005 |
|  | *P. alchemilloides* (L.) L'Hér. | 104 | DEU648PELAR006 |
|  | *P. aridum* R.A. Dyer | 69 | DEU648PELAR008 |
|  | *P. aridum* R.A. Dyer | 106 | DEU648PELAR009 |
|  | *P. frutetorum* R.A. Dyer | 122 | DEU648PELAR031 |
|  | *P. frutetorum* R.A. Dyer | 46 | DEU648PELAR032 |
|  | *P. inquinans* (L.) L'Hér. | 15 | DEU648PELAR046 |
|  | *P. inquinans* (L.) L'Hér. | 128 | DEU648PELAR047 |
|  | *P. multibracteatum* Hochst. ex A. Rich. | 18 | DEU648PELAR055 |
|  | *P. multibracteatum* Hochst. ex A. Rich. | 132 | DEU648PELAR056 |
|  | *P. peltatum* (L.) L'Hér. | 26 | DEU648PELAR066 |
|  | *P. peltatum* (L.) L'Hér. | 44 | DEU648PELAR067 |
|  | *P. peltatum* (L.) L'Hér. | 135 | DEU648PELAR068 |
|  | *P. peltatum* (L.) L'Hér. | 506 | DEU648PELAR069 |
|  | *P. quinquelobatum* Hochst. ex A. Rich. | 138 | DEU648PELAR072 |
|  | *P. tongaense* Vorster | 505 | DEU648PELAR082 |
|  | *P. zonale* (L.) L'Hér. | 33 | DEU648PELAR089 |
|  | *P. zonale* (L.) L'Hér. | 43 | DEU648PELAR090 |
|  | *P. zonale* (L.) L'Hér. | 149 | DEU648PELAR091 |
|  | *P. zonale* (L.) L'Hér. | 504 | DEU648PELAR092 |
|  | *P. zonale* (L.) L'Hér. | 508 | DEU648PELAR093 |
|  | *P. zonale* (L.) L'Hér. | 509 | DEU648PELAR094 |
|  |  |  |  |
|  | **Unassigned species** |  |  |
|  | *P. caylae* Humbert | 47 | DEU648PELAR017 |
|  | *P. caylae* Humbert | 112 | DEU648PELAR016 |
|  | *P. caylae* Humbert | 318 | DEU648PELAR018 |
|  | *P. transvaalense* R. Knuth | 146 | DEU648PELAR083 |
|  |  |  |  |
| ***Pelargonium*** | Section ***Cortusina* (DC.) Harv.** |  |  |
|  | *P. cortusifolium* L'Hér. | 115 | DEU648PELAR020 |
|  | *P. echinatum* Curtis | 10 | DEU648PELAR028 |
|  | *P. echinatum* Curtis | 119 | DEU648PELAR029 |
|  | *P. magenteum* J.J.A. van der Walt | 130 | DEU648PELAR052 |
|  | *P. magenteum* J.J.A. van der Walt | 433 | DEU648PELAR053 |
|  |  |  |  |
|  | Section ***Hoarea* (Sweet) DC.** |  |  |
|  | *P. longifolium* Jacq. | 17 | DEU648PELAR051 |
|  | *P. oblongatum* E. Mey. ex Harv. | 23 | DEU648PELAR06 |
|  |  |  |  |
|  | Section ***Ligularia* (Sweet) Harv.** |  |  |
|  | *P. fulgidum*(L.) L'Hér. | 11 | DEU648PELAR034 |
|  | *P. fulgidum* (L.) L'Hér. | 48 | DEU648PELAR035 |
|  | *P. fulgidum* (L.) L'Hér. | 123 | DEU648PELAR036 |
|  | *P. hirtum* (Burm. f.) Jacq. | 14 | DEU648PELAR045 |
|  |  |  |  |
|  | *Section* ***Magnistipulacea* (R. Knuth) Roeschenbl. & F. Albers** |  |  |
|  | *P. bowkeri* Harv. | 425 | n.d. |
|  | *P. schizopetalum* Sweet | 141 | DEU648PELAR076 |
|  |  |  |  |
|  | Section ***Otidia* (Lindley ex Sweet) DC.** |  |  |
|  | *P. carnosum* (L.) L'Hér. subsp. *carnosum* | 5 | DEU648PELAR015 |
|  | *P. crithmifolium* Sm. | 8 | DEU648PELAR024 |
|  | *P. klinghardtense* R. Knuth | 16 | DEU648PELAR049 |
|  | *P. laxum* (Sweet) G. Don subsp. *laxum* | 129 | DEU648PELAR050 |
|  |  |  |  |
|  | Section ***Pelargonium* (DC.) Harv.** |  |  |
|  | *P. betulinum* (L.) L'Hér. | 501 | DEU648PELAR012 |
|  | *P. betulinum* (L.) L'Hér. | 502 | DEU648PELAR013 |
|  | *P. capitatum* (L.) L'Hér. | 40 | DEU648PELAR014 |
|  | *P. cordifolium* (Cav.) Curtis | 6 | DEU648PELAR019 |
|  | *P. cordifolium* (Cav.) Curtis | 662 | n.d. |
|  | *P. crispum* (P.J. Bergius) L'Hér. | 657 | DEU648PELAR023 |
|  | *P. cucullatum* (L.) L'Hér. subsp.? | 9 | DEU648PELAR025 |
|  | *P. cucullatum* (L.) L'Hér. subsp? | 41 | DEU648PELAR027 |
|  | *P. cucullatum* (L.) L'Hér. subsp? | 118 | DEU648PELAR026 |
|  | *P. fruticosum* (Jacq.) Willd. | 507 | DEU648PELAR033 |
|  | *P. glutinosum* (Jacq.) L'Hér. | 124 | DEU648PELAR037 |
|  | *P. grandiflorum* (Andrews) Willd. | 12 | DEU648PELAR038 |
|  | *P. grandiflorum* (Andrews) Willd. | 125 | DEU648PELAR039 |
|  | *P. graveolens* L'Hér. | 126 | DEU648PELAR040 |
|  | *P. graveolens* L'Hér. | 609 | DEU648PELAR041 |
|  | *P. graveolens* L'Hér. | 666 | DEU648PELAR042 |
|  | *P. panduriforme* Eckl. & Zeyh. | 134 | DEU648PELAR064 |
|  | *P. papilionaceum* (L.) L'Hér. | 25 | DEU648PELAR065 |
|  | *P. quercifolium* (L. f.) L'Hér. | 137 | DEU648PELAR071 |
|  | *P. scabrum* (L.) L'Hér. | 140 | DEU648PELAR075 |
|  | *P. tabulare* (L.) L'Hér. | 29 | DEU648PELAR078 |
|  | *P. tomentosum* Jacq. | 144 | DEU648PELAR080 |
|  | *P. vitifolium* (L.) L'Hér. | 32 | DEU648PELAR085 |
|  | *P. vitifolium* (L.) L'Hér. | 39 | DEU648PELAR087 |
|  | *P. vitifolium* (L.) L'Hér. | 51 | DEU648PELAR086 |
|  |  |  |  |
|  | Section ***Polyactium* DC.** |  |  |
|  | *P. pulverulentum* Colv. ex Sweet | 136 | DEU648PELAR070 |
|  | *P. radulifolium* (Eckl. & Zeyh.) Steud. | 139 | DEU648PELAR073 |
|  | *P. triste* (L.) L'Hér. | 421 | n.d. |

*https://www.bundessortenamt.de/bsa/en/plant-genetic-resources/german-gene-bank-for-ornamentals/
