# Supplementary material for: The variability of nuclear DNA content of different Pelargonium species estimated by flow cytometry
Source: PLoS One. 2022 Apr 28;17(4):e0267496. doi: 10.1371/journal.pone.0267496 (PMC9049363; doi:10.1371/journal.pone.0267496)
Supplement: S2 Table — (DOCX) [file pone.0267496.s002.docx]

**S2 Table. Analysis of intraspecific genome size variation in nine *Pelargonium* species.**

| **Subgenus** | **Species** | **№ JKI Accession** | **Ploidy level (*x*)** | **2C DNA content (pg)*** | **SD** | **№ of samples** |
| --- | --- | --- | --- | --- | --- | --- |
| ***Magnipetala*** | Section ***Myrrhidium*** |  |  |  |  |  |
|  | *P. myrrhifolium* var. *myrrhifolium* | 20 | 2 | 2.16^b^ | 0.10 | 11 |
|  | *P. myrrhifolium* var. *coriandrifolium* | 22 | 2 | 1.55^a^ | 0.05 | 10 |
|  | *P. myrrhifolium* var. *synnotii* | 21 | 2 | 1.53^a^ | 0.07 | 6 |
|  |  |  |  |  |  |  |
| ***Paucisignata*** | Section ***Ciconium*** |  |  |  |  |  |
|  | *P. acetosum* | 1 | 2 | 2.34^a^ | 0.06 | 9 |
|  | *P. acetosum* | 1/7 | 2 | 2.44^b^ | 0.05 | 10 |
|  | *P. acetosum* | 102 | 2 | 2.45^b^ | 0.06 | 8 |
|  |  |  |  |  |  |  |
|  | *P. peltatum* | 26 | 2 | 2.24^a^ | 0.05 | 16 |
|  | *P. peltatum* | 44 | 2 | 2.22^a^ | 0.06 | 17 |
|  | *P. peltatum* | 135 | 2 | 2.19^a^ | 0.05 | 22 |
|  | *P. peltatum* | 506 | 2 | 2.19^a^ | 0.05 | 18 |
|  |  |  |  |  |  |  |
|  | *P. zonale* | 33 | 2 | 2.30^a^ | 0.05 | 4 |
|  | *P. zonale* | 43 | 2 | 2.34^a^ | 0.07 | 3 |
|  | *P. zonale* | 149 | 2 | 2.40^a^ | 0.05 | 4 |
|  | *P. zonale* | 504 | 2 | 2.39^a^ | 0.02 | 3 |
|  | *P. zonale* | 508 | 2 | 2.39^a^ | 0.01 | 3 |
|  | *P. zonale* | 509 | 4 | 4.55^b^ | 0.20 | 6 |
|  |  |  |  |  |  |  |
|  | **Unassigned species** |  |  |  |  |  |
|  | *P. caylae* | 47 | 4 | 4.85^a^ | 0.07 | 4 |
|  | *P. caylae* | 112 | 4 | 4.90^a^ | 0.12 | 7 |
|  | *P. caylae* | 318 | 4 | 5.00^a^ | 0.06 | 5 |
|  |  |  |  |  |  |  |
| ***Pelargonium*** | Section ***Ligularia*** |  |  |  |  |  |
|  | *P. fulgidum* | 11 | 2 | 1.54^a^ | 0.06 | 8 |
|  | *P. fulgidum* | 48 | 2 | 1.55^a^ | 0.05 | 11 |
|  | *P. fulgidum* | 123 | 2 | 1.50^a^ | 0.05 | 8 |
|  |  |  |  |  |  |  |
|  | Section ***Pelargonium*** |  |  |  |  |  |
|  | *P. cucullatum* subsp.? | 9 | 2 | 1.15^b^ | 0.01 | 5 |
|  | *P. cucullatum* subsp? | 41 | 2 | 1.14^b^ | 0.00 | 3 |
|  | *P. cucullatum* subsp? | 118 | 2 | 1.10^a^ | 0.03 | 6 |
|  |  |  |  |  |  |  |
|  | *P. graveolens* | 126 | 8 | 4.09^a^ | 0.06 | 4 |
|  | *P. graveolens* | 609 | 8 | 4.04^b^ | 0.04 | 5 |
|  | *P. graveolens* | 666 | 8 | 3.78^b^ | 0.05 | 3 |
|  |  |  |  |  |  |  |
|  | *P. vitifolium* | 32 | 8 | 4.49^b^ | 0.17 | 6 |
|  | *P. vitifolium* | 39 | 8 | 4.24^b^ | 0.10 | 7 |
|  | *P. vitifolium* | 51 | 8 | 4.22^a^ | 0.03 | 3 |

SD: standard deviation. *Different letters within the species show significant differences, Tukey's b test, α = 5%
