# Supplementary material for: The variability of nuclear DNA content of different Pelargonium species estimated by flow cytometry
Source: PLoS One. 2022 Apr 28;17(4):e0267496. doi: 10.1371/journal.pone.0267496 (PMC9049363; doi:10.1371/journal.pone.0267496)
Supplement: S3 Table — (DOCX) [file pone.0267496.s003.docx]

**S3 Table. Summary of available 2C and 1C*x* DNA contents of *Pelargonium* species reported in this study, by Nieuwenhuis [34] and Weng *et al.* [22].**

| **Species^#^** | **№ JKI Accession^#^** | **Ploidy level*** | **2C DNA content (pg)** | | | **1C*x* DNA content (pg)** | |
| --- | --- | --- | --- | --- | --- | --- | --- |
|  |  |  | **Plaschil *et al.*^1^** | **Nieuwenhuis [34]^2^** | **Weng *et al.* [22]^3^** | **Plaschil *et al.*** | **Nieuwenhuis [34]** |
| Section ***Chorisma*** |  |  |  |  |  |  |  |
| *P. mollicomum* | 131 | 2 | 2.53 |  |  | 1.26 |  |
| *P. tetragonum* | 30 | 2 | 2.91 | 3.09, 2.42, 2.44 | 4.01 | 1.46 | 1.54, 1.21, 1.22 |
| *P. worcesterae* | 72 | 2 | 2.63 |  |  | 1.32 |  |
|  |  |  |  |  |  |  |  |
| Section ***Jenkinsonia*** |  |  |  |  |  |  |  |
| *P. dolomiticum* |  | 2*** |  |  | 1.40 |  |  |
| *P. endlicherianum* |  | 2*** |  |  | 3.95 |  |  |
| *P. mutans* | 133 | 2 | 2.98 | 2.25 |  | 1.49 | n.a. |
| *P. mutans* | 316 | 2 | 2.74 |  |  | 1.37 |  |
| *P. praemorsum* |  | n.a. |  | 5.43 |  |  | n.a. |
| *P. trifidum* | 147 | 2 | 2.37 |  | 1.78 | 1.18 |  |
|  |  |  |  |  |  |  |  |
| Section ***Myrrhidium*** |  |  |  |  |  |  |  |
| *P. caucaulifolium* |  | 4** |  | 2.97, 2.91, 2.93 |  |  | 0.74, 0.73, 0.73 |
| *P. myrrhifolium* var. *myrrhifolium* | 20 | 2 | 2.16 |  |  | 1.08 |  |
| *P. myrrhifolium* (var. *coriandrifolium*) | 22 | 2 | 1.55 |  | 1.29 | 0.77 |  |
| *P. myrrhifolium* var. *synnotii* | 21 | 2 | 1.53 |  |  | 0.76 |  |
| *P. suburbanum* |  | 2** |  | 1.89 |  |  | 0.94 |
|  |  |  |  |  |  |  |  |
| **Unassigned species** |  |  |  |  |  |  |  |
| *P. antidysentericum* |  |  |  | 2.67 |  |  | n.a. |
|  |  |  |  |  |  |  |  |
| Section ***Isopetalum*** |  |  |  |  |  |  |  |
| *P. cotyledonis* | 74 | 2 | 0.94 | 0.98 | 0.68 | 0.47 | 0.49 |
| *P. cotyledonis* | 116 | 2 | 0.92 |  |  | 0.46 |  |
|  |  |  |  |  |  |  |  |
| Section ***Peristera*** |  |  |  |  |  |  |  |
| *P. australe* | 109 | 2 | 1.12 |  | 0.63 | 0.56 |  |
| *P. grossularioides* | 13 | 2, **4** | 2.57 | 2.48^d^ | 1.81 | 0.64 | 1.24^d^ |
| *P. litorale* |  |  |  | 1.07 |  |  | n.a. |
| *P. nanum* |  |  |  |  | 0.75 |  |  |
| *P. rodneyanum* | 65 |  | 1.31 |  |  | n.a. |  |
|  |  |  |  |  |  |  |  |
| Section ***Reniformia*** |  |  |  |  |  |  |  |
| *P. abrotanifolium* | 101 | 2, **4** | 3.35 |  |  | 0.84 |  |
| *P. dichondrifolium* |  | 2, 4*** |  | 1.50 | 1.08 |  | n.a. |
| *P. exstipulatum* |  | 2** |  | 1.39, 1.50 | 1.12 |  | 0.70, 0.75 |
| *P. ionidiflorum* | 73 | 2 | 1.60 | 1.49, 1.53 |  | 0.80 | 0.75, 0.77 |
| *P. odoratissimum* | 52 | 2 | 1.74 | 1.53 |  | 0.87 | n.a. |
| *P. odoratissimum* | 432 | 2 | 1.66 |  |  | 0.83 |  |
| *P. reniforme* subsp. *reniforme* | 28 | **2**, 4 | 1.69 | 3.05^t^ | 1.14 | 0.85 | 0.76^t^ |
| *P. sidoides* | 142 | 2, **4** | 3.58 |  |  | 0.90 |  |
| *P. sidoides* | 321 | 2, 4 | 6.39 |  |  | (0.80 ≙ **8*x***) |  |
|  |  |  |  |  |  |  |  |
| Section ***Ciconium*** |  |  |  |  |  |  |  |
| *P. acetosum* | 1 | 2 | 2.34 | 2.36 |  | 1.17 | 1.18 |
| *P. acetosum* | 1/7 | 2 | 2.44 |  |  | 1.22 |  |
| *P. acetosum* | 102 | 2 | 2.45 |  |  | 1.22 |  |
| *P. acraeum* | 103 | 2 | 2.47 | 2.29 |  | 1.24 | 1.14 |
| *P. alchemilloides* | 2 | 2, **4** | 4.23 | 2.15^d^ |  | 1.06 | 1.08^d^ |
| *P. alchemilloides* | 104 | 2, **4** | 4.24 |  |  | 1.06 |  |
| *P. aridum* | 69 | 2 | 2.38 | 2.19 |  | 1.19 | 1.10 |
| *P. aridum* | 106 | 2 | 2.34 |  |  | 1.17 |  |
| *P. frutetorum* | 122 | 2 | 2.32 |  |  | 1.16 |  |
| *P. frutetorum* | 46 | 2 | 2.40 |  |  | 1.20 |  |
| *P. inquinans* | 15 | 2 | 2.44 | 2.26 |  | 1.22 | 1.13 |
| *P. inquinans* | 128 | 2 | 2.37 |  |  | 1.19 |  |
| *P. multibracteatum* | 18 | 4 | 3.59 |  |  | 0.90 |  |
| *P. multibracteatum* | 132 | 4 | 3.57 |  |  | 0.89 |  |
| *P. peltatum* | 26 | **2**, 4 | 2.24 | 4.53^d^, 4.46^d^ |  | 1.12 | 2.26^d^, 2.23^d^ |
| *P. peltatum* | 44 | **2**, 4 | 2.22 |  |  | 1.11 |  |
| *P. peltatum* | 135 | **2**, 4 | 2.19 |  |  | 1.10 |  |
| *P. peltatum* | 506 | **2**, 4 | 2.19 |  |  | 1.10 |  |
| *P. quinquelobatum* | 138 | 2 | 4.54 |  | 3.77 | (1.14 ≙ **4*x***) |  |
| *P. tongaense* | 505 | 2 | 2.77 | 2.54 | 1.93 | 1.38 | 1.27 |
| *P. zonale* | 33 | 2 | 2.30 |  |  | 1.15 |  |
| *P. zonale* | 43 | 2 | 2.34 |  |  | 1.17 |  |
| *P. zonale* | 149 | 2 | 2.40 |  |  | 1.20 |  |
| *P. zonale* | 504 | 2 | 2.39 |  |  | 1.20 |  |
| *P. zonale* | 508 | 2 | 2.39 |  |  | 1.20 |  |
| *P. zonale* | 509 | 4 | 4.55 |  |  | 1.14 |  |
|  |  |  |  |  |  |  |  |
| Section ***Subsucculentia*** |  |  |  |  |  |  |  |
| *P. otaviense* |  | 2** |  | 2.14 |  |  | 1.07 |
| *P. spinosum* |  | 2*** |  |  | 1.72 |  |  |
|  |  |  |  |  |  |  |  |
| **Unassigned species** |  |  |  |  |  |  |  |
| *P. caylae* | 47 | 4 | 4.85 | 3.02 |  | 1.21 | 0.76 |
| *P. caylae* | 112 | 4 | 4.90 |  |  | 1.22 |  |
| *P. caylae* | 318 | 4 | 5.00 |  |  | 1.25 |  |
| *P. karooicum* |  |  |  | 1.55, 4.20 |  |  | n.a. |
| *P. transvaalense* | 146 | 2 | 3.45 | 2.87 | 2.71 | 1.72 | 1.44 |
|  |  |  |  |  |  |  |  |
| Section ***Campylia*** |  |  |  |  |  |  |  |
| *P. ovale* |  | 2** |  | 1.27 |  |  | 0.63 |
|  |  |  |  |  |  |  |  |
| Section ***Cortusina*** |  |  |  |  |  |  |  |
| *P. cortusifolium* | 115 | 2 | 1.15 |  |  | 0.58 |  |
| *P. echinatum* | 10 | 2 | 1.09 |  | 0.86 | 0.54 |  |
| *P. echinatum* | 119 | 2 | 1.06 |  |  | 0.53 |  |
| *P. magenteum* | 130 | 2 | 1.13 |  |  | 0.57 |  |
| *P. magenteum* | 433 | 2 | 1.30 |  |  | 0.63 |  |
|  |  |  |  |  |  |  |  |
| Section ***Hoarea*** |  |  |  |  |  |  |  |
| *P. incrassatum* |  | 2*** |  |  | 1.08 |  |  |
| *P. longifolium* | 17 | 2 | 0.84 |  |  | 0.42 |  |
| *P. oblongatum* | 23 | 2 | 1.77 |  |  | (0.44 ≙ **4*x***) |  |
|  |  |  |  |  |  |  |  |
| Section ***Ligularia*** |  |  |  |  |  |  |  |
| *P. appendiculatum* |  | 2** |  | 0.97 |  |  | 0.48 |
| *P. fulgidum* | 11 | 2 | 1.54 | 1.48, 1.50 | 1.11 | 0.77 | 0.74, 0.75 |
| *P. fulgidum* | 48 | 2 | 1.55 |  |  | 0.78 |  |
| *P. fulgidum* | 123 | 2 | 1.50 | 1.51 |  | 0.75 | 0.76 |
| *P. hirtum* | 14 | **2**, 4 | 1.40 | 1.57 |  | 0.70 | n.a. |
| *P. hystix* |  | 2*** |  |  | 1.26 |  |  |
| *P. stipulaceum* |  | 2** |  | 1.57 |  |  | 0.78 |
|  |  |  |  |  |  |  |  |
| Section ***Magnistipulacea*** |  |  |  |  |  |  |  |
| *P. bowkeri* | 425 | 4 | 2.89 | 2.79^x^ |  | 0.72 | n.a. |
| *P. luridum* |  | 2, 4, 6, 8*** |  |  | 5.08 |  |  |
| *P. schizopetalum* | 141 |  | 6.69 | 6.96 |  | n.a. | n.a. |
|  |  |  |  |  |  |  |  |
| Section ***Otidia*** |  |  |  |  |  |  |  |
| *P. carnosum* | 5 | 2 | 1.30 | 1.48 |  | 0.65 | n.a. |
| *P. ceratophyllum* |  | 2** |  | 1.46 |  |  | 0.73 |
| *P. crithmifolium* | 8 | 4 | 1.37 |  |  | (0.69 ≙ **2x**) |  |
| *P. dasyphyllum* |  |  |  | 2.94 |  |  | n.a. |
| *P. klinghardtense* | 16 | 2 | 1.38 |  |  | 0.69 |  |
| *P. laxum* | 129 | 2 | 1.49 |  |  | 0.75 |  |
|  |  |  |  |  |  |  |  |
| Section ***Pelargonium*** |  |  |  |  |  |  |  |
| *P. betulinum* | 501 | 2 | 1.07 |  |  | 0.53 |  |
| *P. betulinum* | 502 | 2 | 1.05 |  |  | 0.52 |  |
| *P. capitatum* | 40 | 6 | 3.47 |  |  | 0.58 |  |
| *P. cordifolium* | 6 | 2 | 1.06 |  |  | 0.53 |  |
| *P. cordifolium* | 662 | 2 | 1.05 |  |  | 0.52 |  |
| *P. crispum* | 657 | 2 | 1.10 |  |  | 0.55 |  |
| *P. cucullatum*subsp.? | 9 | 2 | 1.15 | 1.16 | 0.83 | 0.58 | 0.58 |
| *P. cucullatum*subsp? | 41 | 2 | 1.14 |  |  | 0.57 |  |
| *P. cucullatum*subsp? | 118 | 2 | 1.10 |  |  | 0.55 |  |
| *P. fruticosum* | 507 | 2 | 1.04 |  |  | 0.52 |  |
| *P. glutinosum* | 124 | 4 | 2.32 |  |  | 0.58 |  |
| *P. grandiflorum* | 12 | 2 | 0.99 | 1.06 |  | 0.49 | 0.53 |
| *P. grandiflorum* | 125 | 2 | 0.95 |  |  | 0.48 |  |
| *P. graveolens* | 126 | 8 | 4.09 |  |  | 0.51 |  |
| *P. graveolens* | 609 | 8 | 4.04 | 3.91, 4.18 |  | 0.50 | 0.49, 0.52 |
| *P. graveolens* | 666 | 8 | 3.78 |  |  | 0.47 |  |
| *P. hispidum* |  | 4** |  | 1.97 |  |  | 0.49 |
| *P. panduriforme* | 134 | 4 | 2.13 |  |  | 0.53 |  |
| *P. papilionaceum* | 25 | 4 | 2.26 |  |  | 0.56 |  |
| *P. quercifolium* | 137 | 4 | 2.31 |  | 1.72 | 0.58 |  |
| *P. radens* |  | 8** |  | 4.29 |  |  | 0.54 |
| *P. scabrum* | 140 | 2 | 1.08 | 1.19 |  | 0.54 | 0.60 |
| *P. tabulare* | 29 | 2 | 1.31 |  |  | 0.66 |  |
| *P. tomentosum* | 144 | 4 | 2.26 |  | 1.81 | 0.56 |  |
| *P. vitifolium* | 32 | 8 | 4.49 | 4.59 |  | 0.56 | 0.57 |
| *P. vitifolium* | 39 | 8 | 4.24 |  |  | 0.54 |  |
| *P. vitifolium* | 51 | 8 | 4.22 |  |  | 0.53 |  |
|  |  |  |  |  |  |  |  |
| Section ***Polyactium*** |  |  |  |  |  |  |  |
| *P. gibbosum* |  | 2** |  | 1.34, 1.38 |  |  | 0.67, 0.69 |
| *P. multiradiatum* |  | 2** |  | 1.72 |  |  | 0.86 |
| *P. pulverulentum* | 136 | **2**, 4, 6 | 0.89 | 2.84^t^ |  | 0.45 | 0.71^t^ |
| *P. radulifolium* | 139 | 6 | 4.29 |  |  | 0.72 |  |
| *P. triste* | 421 | 4, **6** | 4.12 | 3.96^t^, 3.05^t^, 4.18^t^ |  | 0.69 | 0.99^t^, 0.76^t^, 1.04^t^ |
|  |  |  |  |  |  |  |  |
| **Unassigned species** |  |  |  |  |  |  |  |
| *P. desertorum* |  | 2** |  | 1.13 |  |  | 0.57 |
| *P. xerophyton* |  |  |  | 1.01, 2.38, 2.27^x^ |  |  | n.a. |

^#^ Accessions of the *Pelargonium* species analyzed by Plaschil et al., Nieuwenhuis 2013 and Weng et al. 2012 are different.

*Ploidy level from the present paper, in bold the assumed ploidy level of the accession.

**Ploidy level reported by Nieuwenhuis [34].

***Ploidy level reported by Weng et al. [22].

^1^Nuclei stained with PI, internal standards: tomato ‘Stupické’, *Raphanus sativus* or cauliflower 'Korso'.

^2^Most nuclei stained with DAPI, a few with PI, internal standard: *Vinca minor*, values rounded to 2 decimal places after the decimal point, every value is genome size of a different accession.

^3^Nuclei stained with PI, internal standards: *Arabidopsis thaliana* and trout erythrocytes, data of the paired measurements were averaged and reported as 2C-values.

^d^Ploidy level *2x* is reported.

^t^ Ploidy level *4x* is reported.

^x^ average of two measurements of the same accession.
